# A new approach for the large-scale generation of mature dendritic cells from adherent PBMC using roller bottle technology

**DOI:** 10.1186/1476-8518-6-1

**Published:** 2008-03-06

**Authors:** Ryan E Campbell-Anson, Diane Kentor, Yi J Wang, Kathryn M Bushnell, Yufeng Li, Luis M Vence, Laszlo G Radvanyi

**Affiliations:** 1 Department of Melanoma Medical Oncology, University of Texas, M.D. Anderson Cancer Center, Houston, TX, 77030, USA; 2 Department of Breast Medical Oncology, University of Texas, M.D. Anderson Cancer Center, Houston, TX, 77030, USA

## Abstract

**Background:**

Human monocyte-derived DC (mDC) loaded with peptides, protein, tumor cell lysates, or tumor cell RNA, are being tested as vaccines against multiple human malignancies and viral infection with great promise. One of the factors that has limited more widespread use of these vaccines is the need to generate mDC in large scale. Current methods for the large-scale cultivation of mDC in static culture vessels are labor- and time- intensive, and also require many culture vessels. Here, we describe a new method for the large-scale generation of human mDC from human PBMC from leukopheresis or buffy coat products using roller bottles, never attempted before for mDC generation. We have tested this technology using 850 cm^2 ^roller bottles compared to conventional T-175 flat-bottom static culture flasks.

**Methods:**

DC were generated from adherent human PBMC from buffy coats or leukopherisis products using GM-CSF and IL-4 in T-175 static flasks or 850 cm^2 ^roller bottles. The cells were matured over two days, harvested and analyzed for cell yield and mature DC phenotype by flow cytometry, and then functionally analyzed for their ability to activate allogeneic T-cell or recall antigen peptide-specific T-cell responses.

**Results:**

Monocytes were found to adhere inside roller bottles to the same extent as in static culture flasks. The phenotype and function of the mDC harvested after maturation from both type of culture systems were similar. The yield of mDC from input PBMC in the roller bottle system was similar as in the static flask system. However, each 850 cm^2 ^roller bottle could be seeded with 4–5 times more input PBMC and could yield 4–5 times as many mDC per culture vessel than the static flasks as a result.

**Conclusion:**

Our results indicate that the roller bottle technology can generate similar numbers of mDC from adherent PBMC as traditional static flask methods, but with having to use fewer culture vessels. Thus, this may be a more practical method to generate mDC in large-scale cutting down on the amount of laboratory manipulations, and can save both time and labor costs.

## Background

Dendritic cells (DC) are the most potent antigen-presenting cells (APC) in the immune system that are the key cells activating T-cell-based immune responses against viral disease and cancer [1]. Recently, this powerful ability of DC is being tested as an active vaccine approach to treat cancer and viral infections such as HIV and CMV [2,3]. Most of these studies use monocyte-derived DC (mDC) loaded with antigen *in vitro *and then injected subcutaneously or intravenously [4]. The most commonly used method to generate mDC is to adhere monocytes on to plastic in static flasks from PBMC followed by culture with GM-CSF and IL-4 and maturation using any one of a number of cocktails of pro-inflammatory cytokines (IL-1β, TNF-α, IL-6) or Toll-like receptor (TLR) agonists such as LPS [1,2]. Antigen-loaded DC vaccines have been tested in multiple malignancies, including melanoma, breast cancer, prostate cancer, renal cancer, and follicular lymphoma, where they have been found to consistently induce antigen-specific CD4^+ ^and CD8^+ ^T-cell responses along with some reported clinical response [5-7].

The production of DC vaccines requires the cultivation of millions of clinical-grade mDC in large-scale. In some cases more than a billion mDC may be required to ensure that enough vaccine can be produced for multiple patient immunizations over a number of months. Vaccination regimens using antigen-pulsed mDC have ranged from multiple 10–20 × 10^6 ^mDC to up to 100 × 10^6 ^or more mDC injected subcutaneously or intravenously, respectively [8,9]. Monocytes differentiate directly into DC in these cultures and do not divide and, as a result, leukopheresis products containing billions of PBMC are required as starting material to have enough monoytes available for the procedure. Current methods for large-scale cultivation of mDC in static culture systems can be cumbersome, labor- and time- intensive, and require many repetitive culture vessels or multi-layered systems [10-13]. The numerous manipulations required to set-up most static culture flasks for large-scale mDC generation also increases the chances for product variability from culture to culture and sterility being compromised. Although non-adherent cell culture systems of isolated CD14^+ ^monocytes have been introduced, there is still some debate on the quality of these mDC versus those derived from adherent populations. For example, some studies of have found decreased yields of mature CD83^+ ^mDC or reduced IL-12 production capability versus adherent systems [14,15]. Thus, any improvements in the speed and ease of generating DC from adherent monocytes in large scale and better purity for clinical use would be a great asset.

We describe a novel method of generating mature mDC in large-scale using roller bottle culture technology never before reported to be used to generate DC before. The monocytes from the peripheral blood mononuclear cell (PBMC) or leukopheresis preparations were adhered to the inside surface of roller bottles on a roller apparatus at low speed. After removal of the non-adherent cells, DC cells are generated using culture medium containing GM-CSF and IL-4 and matured using any one of a number of well-defined defined cytokine cocktails. This resulted in a large number of floating non-adherent mature DC that can be easily harvested and used for vaccines or other purposes. The roller bottle DC had similar phenotypic and functional characteristics as those produced in static culture flasks. Overall, the roller bottle system is a self-contained system requiring minimal manipulation during culture set-up. The result is faster culture set-up times and less labor for lab personnel than traditional static culture methods in flat-bottom culture flasks.

## Methods

### Reagents and equipment

Human recombinant cytokines (GM-CSF, IL-4, IL-1β, TNF-α, and IL-6) were purchased from R&D Systems (Minneapolis, MN). Prostaglandin E_2 _(PGE_2_) was purchased from Sigma-Aldrich (St. Louis, MO). Dendritic cell culture medium (DC-CM) consisted of Iscove's Modified Dulbecco's Medium (IMDM) containing Glutamax, 20 μg/ml gentamycin, 50 μM 2-mercaptoethanol (all from Invitrogen, Carlsbad, CA), and 2% normal human AB serum (Valley Biomedical, Winchester, VA). Roller bottles (850 cm^2 ^or 490 cm^2^) with vented caps were obtained from Fisher-Costar (Houston, TX). A Stovall Low Profile Roller apparatus (Stovall Life Science Inc., Greensboro, NC) was used for the roller bottle cultures. Flat-bottom static T-175 culture flasks (175 cm^2 ^area) with vented caps were obtained from Nunc (Rochester, NY). All flow cytometry antibodies and 7-aminoactinomycin D (7-AAD) were purchased from BD Biosciences (La Jolla, CA).

### Sources of PBMC for DC generation

PBMC were obtained from peripheral blood leukopheresis products obtained from non-mobilized normal donors (LifeBlood, Memphis, TN), or G-CSF-mobilized normal donors (AllCells, Berkeley, CA). Products were collected in the presence of Anticoagulant Citrate Dextrose Formula A (Gambro). In addition, peripheral blood buffy coats (Gulf Coast Regional Blood Bank, Houston, TX) were also used for some experiments. In some experiments HLA-A*0201^+ ^positive non-mobilized leukopheresis products were used to generate DC (LifeBlood, Memphis, TN). The HLA-A*0201 status was further confirmed by flow cytometry after receipt of the sample in the laboratory. All leukopheresis products and buffy coats were used within 24 hours post-collection. The PBMC were isolated by diluting with HBSS, centrifuged at 400 × g for 20 min over Histopaque-1077 (Sigma-Aldrich). The interface cells were collected, pooled, and washed with HBSS until the contaminating platelets were removed. PBMC not used immediately were frozen in human AB serum with 10% DMSO (33.3 × 10^6^) cells/ml and stored in the vapor phase of liquid nitrogen.

### Dendritic cell culture in roller bottles

Washed PBMC from leukopheresis products or buffy coats were diluted to 30 × 10^6 ^cells/ml in DC-CM and 30 ml (900 × 10^6 ^cells) were seeded into 850 cm^2 ^roller bottles with vented caps (Fisher-Costar, Houston, TX). The bottles were placed on the roller bottle apparatus in a 37°C, 5% CO_2 _incubator and rolled at low speed (1 rpm) for 2 to 3 h. The bottles were then taken out and agitated to loosen any non-adherent cells and the floating cells removed. The bottles were then washed 2 times with 80–100 ml warm DC-CM by rolling the bottle inside a laminar flow hood. After removal of the second wash, 150–180 ml of DC-CM containing 1,000 U/ml GM-CSF and 1,000 U/ml IL-4 was added to each bottle. The bottles were placed back on the roller bottle apparatus in the incubator and rolled at 2 rpm for 4–5 days. A dendritic cell maturation cocktail consisting of a final concentration of 10 ng/ml IL-1β, 10 ng/ml TNF-α, 15 ng/ml IL-6, and 1 μg/ml PGE_2 _(ITIP) [13,16]. After 20–24 h the floating cells were harvested in all bottles and analyzed for mature DC content. In some experiments, an alternative maturation cocktail called the "Pittsburgh Protocol" (25 ng/ml IL-1β, 50 ng/ml TNF-α, 1,000 U/ml IFN-γ, 20 μg/ml poly I:C, and 3,000 U/ml IFN-α) was used to generate so-called α Type-1DC (α DC1) was added on day 4 or 5 [17]. In some experiments, 450 cm^2 ^roller bottles (Fisher-Costar) were used with PBMC seeded at 250 to 450 × 10^6 ^cells per bottle.

### Dendritic cell generation in flat-bottom static T-175 flasks

Washed PBMC from leukopheresis products or buffy coats were seeded into T-175 culture flasks in 15 ml of DC-CM (175 × 10^6 ^cells per flask). The flasks were incubated as above for 2 to 3 h and non-adherent cells were removed. The flasks were then washed with 50 ml of warm DC-CM and 60 ml of DC-CM containing 800 U/ml GM-CSF and 1,000 U/ml IL-4 was added. The cells were incubated for 4–5 days and matured for 20–24 h and analyzed for mature DC content and function as above.

### Determination of mDC yield and phenotype

Isolated cells were washed in DC-CM and viable cell recovery determined with Trypan Blue staining and counting live cells on a hemocytometer using a light microscope. The total floating cells isolated were divided by the number of culture vessels to determine the yield per flask or per bottle. For cell surface staining, the cells were washed 2 times in cold FACS Wash Buffer (FWB) consisting of D-PBS, 1% BSA and re-suspended at 10 × 10^6^/ml in cold FACS Stain Buffer (FSB) consisting of D-PBS, 1% BSA, and 5% normal goat serum. The cells were stained using anti-CD83-PE, anti-CD80-FITC, anti-CD86-APC, CD11c-FITC and CD14-PE (all from BD Biosciences, La Jolla, CA) on ice for 20 min and washed with cold FWB and re-suspended in 0.35 ml cold FWB. 7-AAD (2 μg/ml) was added 5–10 min before FACS analysis to exclude dead cells and enumerate mDC viability. The samples were run on a FACScalibur or FACScanto flow cytometer and analyzed using FlowJo 7.2.2 software (Tree Star Inc., Ashland, OR).

### Functional analysis of isolated mDC

DC isolated from roller bottles and static flask cultures were assayed for their ability to induce allo-antigen T-cell responses and CD8^+ ^T-cell recall responses against HLA-A2-binding epitopes from flu, CMV, and EBV [18]. For allo-antigen responses, 50,000 monocyte-depleted PBMC (2-hour plastic-non-adherent PBMC) from a normal donor other than that used to generate the DC were incubated in U-bottom 96-well plates with different numbers of DC or PBMC stimulators (50,000, 25,000, 10,000, 5,000, 1,000, 500, 200, or 100 cells). On day 6, 1 μCi/well of ^3^H-thymidine was added to each well and the cells harvested the next day and total cpm/well determined. Recall antigen CD8^+ ^T-cell responses were done in ELISPOT plates (Millipore) using 5 × 10^5 ^monocyte-depleted autologous PBMC incubated with peptide-pulsed mDC harvested from roller bottles or static flask cultures. The mDC were pulsed with 5 μg/ml of the HLA-A2-binding epitopes from influenza A matrix (GILGFVFTL), CMV pp65 (NLVPMVATV), and EBV BMLF1 (GLCTLVAML) for 90 min, washed and added to the responder cells in the ELISPOT plates [18]. The plates were incubated overnight and processed as described before [19].

## Results

### Monocytes adhere similarly in roller bottles and static flasks

We first tested whether human monocytes can adhere inside roller bottles as in traditional static flat-bottom flasks. PBMC from normal donor buffy coats were seeded into 490 cm^2 ^roller bottles or T-175 culture flasks and adhered for 2.5 h (1 rpm for the roller bottles) in the incubator. The non-adherent cells were collected and stained for CD14 and CD3 expression. Adherence of monocytes will deplete the CD14^+ ^population in the non-adherent cell suspension. As shown in Table 1, the CD14^+ ^monocytes adhered in roller bottles with similar efficiency as flat-bottom T-175 flasks, as indicated by the drop in percentage of CD14^+ ^cells in the suspended cell fraction.

**Table 1:** Adherence of peripheral blood CD14^+ ^monocytes to roller bottles and static flasks*

**Condition**	**CD14+ (%)**	**CD3+ (%)**	**CD14- and CD3- (%)**
Pre-adherent PBMC	14.6	45.4	40
Flask: Post-adherence	2	59.5	38.4
Roller bottle #1: Post-adherence	2.2	55.2	36.4
Roller bottle #2: Post-adherence	2.1	56	41.9

*PBMC isolated from a normal donor buffy coat donor were incubated in T-175 culture flask or 450 cm^2 ^roller bottles for 2.5 h. The roller bottles were rolled at low speed (1 rpm). The non-adherent cells were isolated and stained along with a sample of the original PBMC for CD14 and CD3 expression. The percent CD14^+^, CD3^+^, or CD14^-^CD3^- ^cells are shown before (pre-adherent PBMC) and after the adherence protocol.

### Similar degree of DC maturation in roller bottles as in static flasks

**Figure 1:**
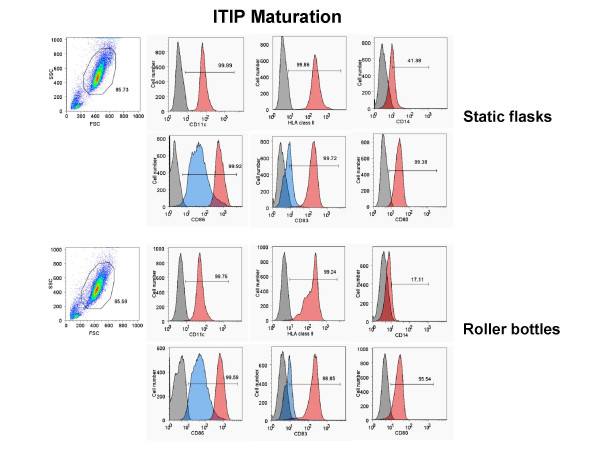
**Generation of phenotypically mature mDC from adherent monocytes using ITIP in roller bottle cultures in comparison to static flask cultures**. PBMC from a normal donor leukopheresis product was seeded into 850 cm^2 ^roller bottles or into T-175 flasks and the monocytes adhered for 2.5 h as described in the Methods section. After washing out the non-adherent cells in both systems, the cells were cultured for 4 days with 1,000 U/ml GM-CSF and 1,000 U/ml IL-4 and then matured using ITIP. The floating cells were harvested after 24 h and stained for CD11c, CD14, HLA class II DP, DQ, DR, CD83, CD86, and CD80. The unstained and stained populations in the histograms are shown in grey and red, respectively. In the case of CD86 and CD83 staining, the surface expression on cells from non-matured cultures (in blue) is shown as a comparison to verify that maturation was induced in both systems. The results of one out of 3 similar experiments are shown.

**Figure 2:**
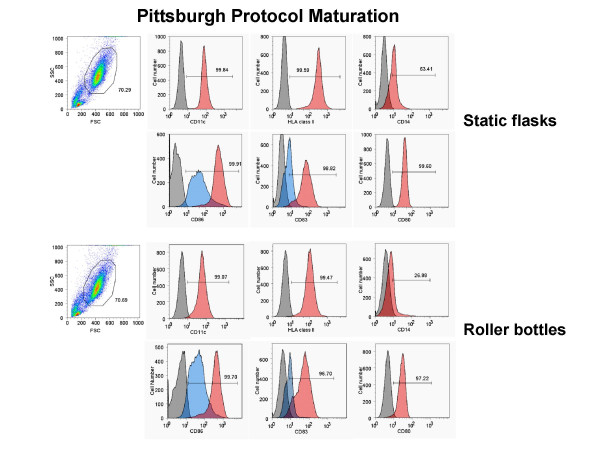
**Generation of phenotypically mature mDC from adherent monocytes using the Pittsburgh Protocol in roller bottle cultures in comparison to static flask cultures**. PBMC from a normal donor leukopheresis product was seeded into 850 cm^2 ^roller bottles or into T-175 flasks and the monocytes adhered for 2.5 h as described in the Methods section. After washing out the non-adherent cells in both systems, the cells were cultured for 4 days with 1,000 U/ml GM-CSF and 1,000 U/ml IL-4 and then matured using the Pittsburgh Protocol combination of cytokines. The floating cells were harvested after 24 h and stained for CD11c, CD14, HLA class II DP, DQ, DR, CD83, CD86, and CD80. The unstained and stained populations in the histograms are shown in grey and red, respectively. In the case of CD86 and CD83 staining, the surface expression on cells from non-matured cultures (in blue) is shown as a comparison to verify that maturation was induced in both systems. The results of one out of 3 similar experiments are shown.

**Figure 3:**
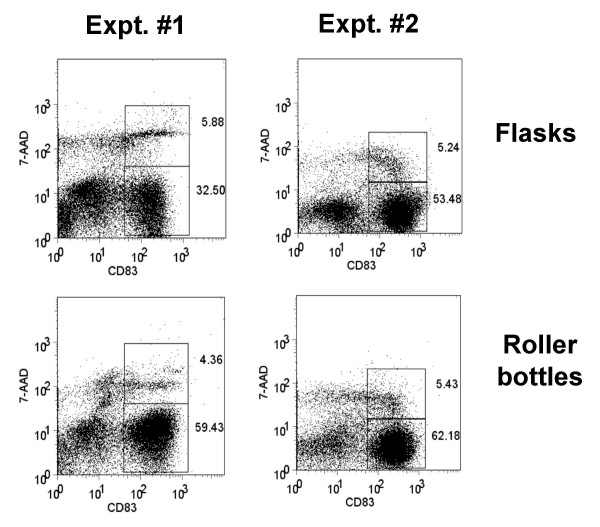
**Roller bottle cultures yield mature CD83^+ ^DC with high viability**. Mature mDC were generated in 850 cm^2 ^roller bottles or in T-175 static flasks as before using ITIP maturation. The floating cells were harvested and stained for DC maturation markers without fixation. Immediately before FACS analysis 2 μg/ml 7-ADD was added as viability indicator. The dot plots shown the total cells in the floating fractions with the CD83^+^, 7-AAD^- ^and CD83^+^, 7-AAD^+ ^cells gated. In both cases, the CD83^+ ^cells exhibited 94–96% viability. The results of two separate experiments are shown.

Next, we generated monocyte-derived DC in 850 cm^2 ^roller bottles versus T-175 static flasks after monocyte adherence and assessed the phenotype and viability of the DC generated from each culture type after maturation with 10 ng/ml IL-1β, 10 ng/ml TNF-α, 15 ng/ml IL-6, and 1 μg/ml PGE_2 _(ITIP). The floating cells isolated from both culture types 24 h after addition of the maturation cocktail were stained for CD83, CD86, CD80, CD11c, and CD14 and analyzed by FACS. Both types of cultures induced comparable levels of DC maturation, as indicated by the similar percentages of CD83^+^, CD80^+^, CD86^hi^, CD11c^+^, CD14^-/lo ^generated using two separate methods, ITIP maturation (Fig. 1) and Pittsburgh Protocol maturation (Fig. 2). The viability of the harvested mature DC from the roller bottles and static flasks was also assessed using 7-AAD staining of the cells prior to FACS analysis. In both cases, the CD83^+ ^DC were > 90% viable, as shown in the two separate experiments shown in Fig. 3.

### Efficiency in generating large numbers of mature DC in roller bottles

One of the benefits of using culture vessels with increased surface area such as roller bottles is maximizing the scale in which DC cells can be generated while minimizing the number of separate culture vessels needed to achieve high-throughput production. Using 850 cm^2 ^roller bottles we found that up to 900 × 10^6 ^PBMC could be loaded during the monocyte adherence step, while up to 180 × 10^6 ^cells could be loaded in T-175 flasks. We determined the yield of total floating cells and mature DC recovered in both culture systems. In these experiments, PBMC from G-CSF-mobilized or non-mobilized leukopheresis products were loaded into the culture vessels and adherent cells treated with GM-CSF and IL-4 for 4 to 5 days followed by treatment with the ITIP maturation cocktail or α DC1 maturation cocktail (not shown) for 24 h. Table 2 shows the results of three separate experiments comparing the yield of mature DC in both systems and the percentage of mature DC relative to the original PBMC load. On average, from normal donor non-GSF-mobilized leukopheresis products a single 850 cm^2 ^roller bottle culture yielded 80–85 × 10^6 ^CD83^+ ^CD86^hi ^DC and the static T-175 flasks yielded up to 10–20 × 10^6^CD83^+ ^CD86^hi ^DC; this represent an average 5 to 6-fold more mDC per single culture vessel (Table 2). In addition, DC from both types of cultures were able to be cryopreserved in 90% AB serum, 10% DMSO with > 80% viability after thawing (data not shown). The roller bottle system could also generate mDC from G-CSF-mobilized leukopheresis products with a similar yield as static flasks (Table 2; Experiment #3). In this case, the yield of mDC per input cells was lower because of the lower percentage of mature CD14^+ ^monocytes in these products than in non-mobilized PBMC. Similar relative results were obtained with the Pittsburgh Protocol maturation protocol (data not shown). Thus, the roller bottle approach allows for the efficient scale-up for the generation of large numbers of mature DC with similar yield of mDC per input cells as in static flasks.

**Table 2:** Yield of mature DC from roller bottle cultures and static flask cultures*

**Expt #**	**Culture system**	**PBMC seeded per vessel**	**Average floating cells recovered**	**Average mature DC (CD83^+^, CD86^hi^) recovered**	**% yield of mature DC**
1**	850 cm^2 ^roller bottles	900 × 10^6^	106 × 10^6^	85 × 10^6^	9.4%
	T-175 static flasks	180 × 10^6^	20 × 10^6^	19.2 × 10^6^	10.7%
2**	850 cm^2 ^roller bottles	900 × 10^6^	100 × 10^6^	82 × 10^6^	9.1%
	T-175 static flasks	180 × 10^6^	16 × 10^6^	10.2 × 10^6^	5.7%
3***	850 cm^2 ^roller bottles	800 × 10^6^	84 × 10^6^	23 × 10^6^	2.9%
	T-175 static flasks	200 × 10^6^	29 × 10^6^	6 × 10^6^	3%

*Human peripheral blood leukopheresis products were seeded and monocytes adhered in the two types of culture vessels, as described in the Methods section. After 4 to 5 days of culture with GM-CSF and IL-4, ITIP cocktail was added to induce DC maturation. On average, 2–3 roller bottles or static flasks were set up for each experiment. The floating cells were harvested one day later and viable cellrecovery was determined by Trypan Blue staining with a hemocytometer followed by FACS staining for CD83 and CD86. The number of mature DC was calculated from the percent CD83^+^, CD86^hi ^cells using the total number of viable floating cells. The results of three separate experiments are shown.

**Experiment was done directly with normal donor non-G-CSF-mobilized leukopheresis products.

***Experiment was done directly with a G-CSF-mobilized normal donor leukopheresis product, accounting for the lower yield of mature DC.

**Figure 4:**
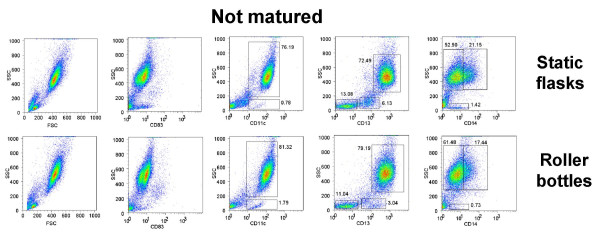
**Phenotypic analysis of DC purity in non-matured DC cultures from roller bottle and static flask cultures**. DC were generated as before in 850 cm^2 ^roller bottles or T-175 static flasks for 4 days and then incubated for an additional 24 h without any additional cytokines ("Not matured"). The floating cells were harvested after this additional 24 h incubation and stained for CD11c, CD13, CD14, CD83 and CD86 and analyzed by flow cytometry. In each case all the isolated floating cells were analyzed without gating and phenotype of DC compared between the roller bottles and static flask system. The numbers in the dot plots indicate the percentage of cells out of the total population of floating cells having the indicated phenotype. The results of one out of 4 similar experiments are shown.

**Figure 5:**
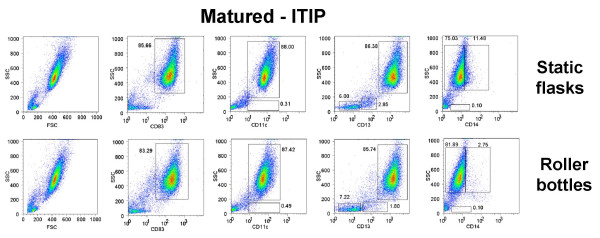
**Phenotypic analysis of DC purity in matured DC cultures from roller bottle and static flask cultures**. DC were generated as before in 850 cm^2 ^roller bottles or T-175 static flasks for 4 days and then incubated for an additional 24 h with ITIP cocktail to induce DC maturation ("Matured-ITIP"). The floating cells were harvested after 24 h after addition of the ITIP maturation cocktail and stained for CD11c, CD13, CD14, CD83 and CD86 and analyzed by flow cytometry. In each case all the isolated floating cells were analyzed without gating and phenotype of DC compared between the roller bottles and static flask system. The numbers in the dot plots indicate the percentage of cells out of the total population of floating cells having the indicated phenotype. The results of one out of 4 similar experiments are shown.

The generation of DC from adherent PBMC from peripheral blood does not yield a 100% pure population of floating mature DC. The mDC are mixed with other cells that are carried over from the original PBMC loaded into the culture vessels during the monocyte adherence step. We determined the percentage of CD83^+^, CD11c^+^, CD13^+^, and CD14^+ ^in the high forward scatter (FSC^hi^) and high side scatter (SSC^hi^) population (DC gate), as well as the low forward scatter (FSC^lo^) and low side scatter (SSC^lo^) population isolated from non-matured (Fig. 4) and ITIP-matured (Fig. 5) cultures from both the static flask and roller bottle systems. The flow cytometry profiles in Fig. 4 and Fig. 5 are all on total (ungated) cells in each sample. CD83 was highly induced in the FSC^hi^, SSC^hi ^population in the ITIP matured cultures from both the flask and roller bottle systems (Fig. 5), while none of FSC^lo^, SSC^lo ^cells expressed these high CD83 levels (Fig. 5). In addition, only the FSC^hi^, SSC^hi ^population was CD11c^+ ^in the matured cultures from both system, with only a small fraction (< 1%) of FSC^lo^, SSC^lo ^cells expressing CD11c (Fig. 5). The FSC^lo^, SSC^lo ^cells were further analyzed and found to consist largely of CD13^-^, CD14^- ^(non-myeloid origin) and CD11c^- ^cells which by process of elimination are essentially lymphocytes (T and B cells) or NK cells. Some FSC^lo^, SSC^lo ^cells having low CD13 expression were found in the non-matured cultures (Fig. 4), but these largely disappeared in the ITIP-matured cultures (Fig. 5) with mostly a minor population CD13^-^, CD14^-^, CD11c^- ^population making up the FSC^lo^, SSC^lo ^population. In the matured cultures from both the flasks and roller bottles, the FSC^lo^, SSC^lo^, CD13^- ^subset was less than 10% in each case (Fig. 5). Lastly, CD14 was down-modulated in cells obtained from both ITIP-matured static flasks and roller bottles (Fig. 5), as compared to cells isolated from non-matured cultures (Fig. 4).

Thus, both roller bottle and static flask cultures yielded mature DC of similar purity with a similar minor population of FSC^lo^, SSC^lo ^cells having a lymphocyte (CD11c^-^, CD13^-^, CD14^-^) phenotype.

### DC generated in roller bottles function similarly as those from static flasks

**Figure 6:**
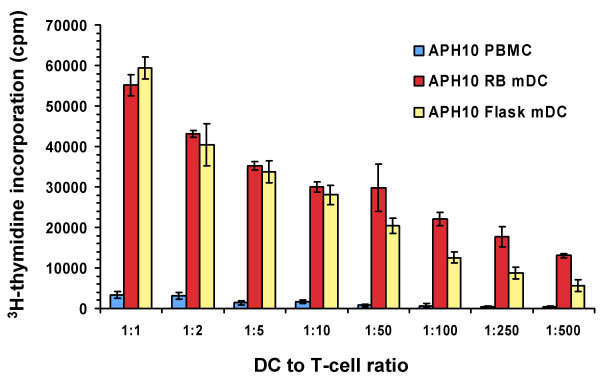
**Dendritic cells generated in roller bottle cultures have potent allo-stimulatory capability**. Dendritic cells were generated in 850 cm^2 ^roller bottles or T-175 static flasks as before using ITIP maturation. The floating cells were harvested, irradiated at 20 Gy and mixed with 50,000 allogeneic T-cell-enriched PBMC (plastic non-adherent PBMC) at different stimulator to responder ratios in 96-well plates. After 5 days, 1 μCi/well of ^3^H-thymidine was added to each well and the plates harvested 18 h later. Irradiated PBMC from the original DC donor were also used as stimulators as a control. The average cpm and standard deviation of triplicate cultures are shown for each stimulator type.

**Figure 7:**
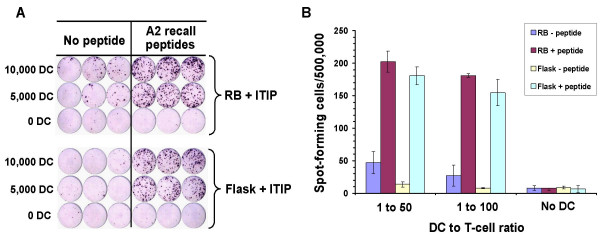
**Dendritic cells generated in roller bottles stimulate autologous peptide-specific T-cell responses**. Dendritic cells from HLA-A*0201^+ ^normal donor leukopheresis products were differentiated and matured with ITIP in roller bottles or static flasks as before. The floating cells were harvested, pooled, irradiated (20 Gy), and pulsed with HLA-A*0201 epitopes from flu, EBV, and CMV (see Materials and Methods for details). The DC were washed and added to 500,000 T-cell-enriched autologous PBMC in anti-IFN-γ antibody-coated ELISPOT plates in the numbers indicated. Each assay was run in triplicate. The plates were harvested after overnight culture and developed. Shown is the image taken of the developed ELISPOT plate (**A**) and corresponding graphical representation of the number of spots per 500,000 input responder cells under the different conditions (**B**). Dendritic cells without peptide pre-pulsing were used as controls. The results are representative of two similar experiments.

In order to determine whether DC generated in roller bottles functioned similarly as antigen-presenting cells (APC) as those generated in static flasks, we tested both types of DC for their ability to activate allo-specific and autologous recall antigen peptide-specific T cell responses. DC were generated in 850 cm^2 ^roller bottles or T-175 static culture flasks as before and the floating cells were isolated and tested for APC activity. Fig. 6 shows an example of the allo-stimulatory function of DC generated from a normal leukopheresis donor (APH 10) in roller bottles versus static flasks. In both cases, the DC induced a similar rate of allo-specific T-cell proliferation at the different DC doses used in the assay (Fig. 6). Floating cells isolated from non-matured roller bottle DC cultures as well as the original PBMC population has substantially lower allo-stimulatory activity on a per cell basis than the mature DC (Fig. 6). In another experiment, we found that both the ITIP and α DC1 maturation protocols in roller bottles induced DC of comparable potent allo-stimulatory capacity in comparison to the original starting PBMC population in the leukopheresis product (data not shown). To test the ability of DC to present peptides and activate autologous T cells, we used a recall antigen response assay using HLA-A*0201-binding peptides. In this case, we generated DC in 850 cm^2 ^roller bottles or T-175 static flasks from HLA-A*0201^+ ^donor leukopheresis products. The floating DC after the maturation step were isolated and pulsed with 9-mer peptides from flu, CMV, and EBV (see Materials and Methods). The peptide-pulsed DC were washed and incubated with autologous monocyte-depleted PBMC in an overnight IFN-γ ELISPOT assay (1:50 or 1:100 DC to responder ratios). As shown in Fig. 7, mature DC generated using either approach yielded cells of comparable APC activity in terms of the number of IFN-γ spot-forming cells in the assay. Little or no IFN-γ production was found in cultures with non-preloaded DC, or in cultures without added DC (Fig. 7). Thus, DC generated in roller bottles yield highly competent APC for T-cell stimulation.

## Discussion

The generation of large numbers of mature DC in a large-scale culture processes for application in vaccine clinical trials still remains a challenge using present static flask technology due to the high number of culture vessels needed. Although new devices such as multi-level static culture devices such as Cell Factories™ (Nunc, Rochester, NY) have improved our ability to generate DC in large scale, most static culture systems are still cumbersome and labor intensive [4,11,13]. We have developed an alternative approach for the large-scale generation of mature DC from adherent human monocytes using roller bottle technology. This system can generate DC from plastic-adherent monocytes as traditional static flask cultures. The DC generated using roller bottles had the same phenotypic and functional attributes as those generated in static flask cultures. However, given the large surface area in a single roller bottle (850 cm^2^), this technology allows for the loading of much higher numbers of input PBMC per single vessel with a comparable level of monocyte adherence and mature DC yield, thereby generating much higher numbers of DC per vessel. A number of benefits arise out of this approach, including the need for up to 5 times less culture vessels to generate the equal number of DC versus static T-175 flasks. The roller bottle method is also easy to perform, more practical than handling large numbers of flasks, and overall saves technician time and potential labor costs. In addition, the less manipulation required to generate DC products in large scale will also help ensure less chance of error and contamination with infectious agents that would destroy the product.

In our initial experiments, we found that monocytes had a similar capacity to adhere to the plastic inside the roller bottles as in the static flasks. Initially, this was a surprise to us, considering the dogma in the field that has emerged with the use of static culture flasks to generate DC for over 15 years. However, in our loading step the PBMC are rolled in the bottles at sufficiently low speed (1 rpm) allowing the monocytes to adhere just as well as in static flasks. The low volume per vessel surface area used during the loading process in the bottles allows the monocytes to roll along and stay in close contact with the surface and then eventually attach. This is akin to the attachment of monocytes rolling along the walls of blood vessels in the body during extravasation into tissues.

Recently, newer static flat-bottom culture systems for DC have been developed such as the Cell Factories™ (Nunc, Rochester, NY) [11,13]. These systems consist of two or more flat-bottom culture surfaces stacked on top of each other in a single large flask format. The cells are seeded into a main port and distributed over the multiple stacked surface areas. We have found however that these vessels are cumbersome to handle and it is not straightforward to evenly distribute the cell and culture medium over all the stacked surfaces in the culture vessel (unpublished observations). In addition, feeding additional growth factors and DC maturation agents so that they are evenly distributed in each culture level also requires additional manipulation and is not straightforward. In contrast, the roller bottle system offers a simpler and more fool-proof method to generate the same or even greater number of mature DC allowing even novice technicians to set-up the cultures with ease and higher reproducibility.

Roller bottles have been used in vaccine manufacture to culture strongly adherent fibroblast producer lines and have never been tested for their ability to generate monocyte-derived DC in large scale. Thus, this approach is a novel application that increases the versatlity of this technology and broadens its application in vaccine manifacturing. In addition, the mDC generated in roller bottles are functionally equivalent in terms of their ability to activate T-cell responses as mDC generated in static flasks.

## Conclusion

The roller bottle method described here is an new and more practical way to generate large numbers of mature DC with potent APC activity for vaccine applications or large scale laboratory studies where > 100 million mDC are routinely needed. The method is easy to perform, saves time, and generates mDC of equal potency and similar purity as traditional static flask methods. The method can also be easily translated into a GMP environment where roller bottles have been used for other applications.

## Competing interests

The author(s) declare that they have no competing interests.

## Authors' contributions

REC performed experiments and wrote the manuscript. DK, YJW, YF, and LMV helped perform experiments. KMB processed and provided human PBMC materials for experiments. LGR supervised the work and helped write the manuscript.
